# The Performance of Several Docking Programs at Reproducing Protein–Macrolide-Like Crystal Structures

**DOI:** 10.3390/molecules22010136

**Published:** 2017-01-17

**Authors:** Alejandro Castro-Alvarez, Anna M. Costa, Jaume Vilarrasa

**Affiliations:** Organic Chemistry Section, Facultat de Química, Diagonal 645, Universitat de Barcelona, 08028 Barcelona, Catalonia, Spain; alecastro@ub.edu (A.C.-A.); amcosta@ub.edu (A.M.C.)

**Keywords:** macrolides, natural products, docking, AutoDock, Vina, DOCK, Glide

## Abstract

The accuracy of five docking programs at reproducing crystallographic structures of complexes of 8 macrolides and 12 related macrocyclic structures, all with their corresponding receptors, was evaluated. Self-docking calculations indicated excellent performance in all cases (mean RMSD values ≤ 1.0) and confirmed the speed of AutoDock Vina. Afterwards, the lowest-energy conformer of each molecule and all the conformers lying 0–10 kcal/mol above it (as given by Macrocycle, from MacroModel 10.0) were subjected to standard docking calculations. While each docking method has its own merits, the observed speed of the programs was as follows: Glide 6.6 > AutoDock Vina 1.1.2 > DOCK 6.5 >> AutoDock 4.2.6 > AutoDock 3.0.5. For most of the complexes, the five methods predicted quite correct poses of ligands at the binding sites, but the lower RMSD values for the poses of highest affinity were in the order: Glide 6.6 ≈ AutoDock Vina ≈ DOCK 6.5 > AutoDock 4.2.6 >> AutoDock 3.0.5. By choosing the poses closest to the crystal structure the order was: AutoDock Vina > Glide 6.6 ≈ DOCK 6.5 ≥ AutoDock 4.2.6 >> AutoDock 3.0.5. Re-scoring (AutoDock 4.2.6//AutoDock Vina, Amber Score and MM-GBSA) improved the agreement between the calculated and experimental data. For all intents and purposes, these three methods are equally reliable.

## 1. Introduction

Many important drugs and hundreds of natural products with promising bioactivities are macrolides or have macrolide-related structures. Such compounds often show favorable pharmacokinetic profiles with regard to open-chain molecules, despite not always following Lipinski’s rules [1]. Conformational search is important for identifying their bioactive conformers, which is a prerequisite for good results in a wide range of modeling methods. Indeed, adequate sampling is extremely important in docking calculations and small changes in the search parameters can have a significant effect on their performance [2].

In this context, over the past two decades our research group has worked on the total synthesis of bioactive macrolide-like natural products, such as fluvirucins [3,4]; amphidinolides X [5], Y [6], K [7], E [8] and B2 [9]; and palmerolide A [10]. We performed docking calculations and collaborated with biochemists, chiefly with the aims of elucidating the mechanisms of action of some cytotoxic macrolides [11,12,13,14,15]. Since then, updated and new docking software has been released [16,17,18,19,20,21,22,23,24,25,26,27]. This led us to wonder whether some explanations and predictions, included in PhD theses or articles in the past decade, would still stand in the present scenario. For example, an updated version of AutoDock 3.0.5 [28] (henceforward AD 3.0, a popular program that we used years ago) is AutoDock 4.2.6 [29] (henceforward AD 4.2). A faster program, AutoDock Vina 1.1.2 [30] (henceforward AD Vina) was more recently launched by the same institution. It is expected that these programs will provide improved results and allow better explanations than those facilitated by AD 3.0. Last year, once most of our AD Vina results were at hand, PSOVina, a program that includes a particle swarm optimization algorithm, was released. PSOVina reduces the calculation time of AD Vina even further; since the accuracy is the same [31], only a few tests have been performed using it. All these packages are free. Other docking methods that we have examined are DOCK 6.5 (henceforward, DOCK) [32] and Glide 6.6 (Glide) [33]. DOCK is also free. Glide, from Schrödinger, appears to be the most popular of the commercial docking programs, according to an exhaustive SciFinder search. Thus, in the present study, we restricted ourselves to these five programs. Comparative studies of different docking programs have been reported involving small molecules (not discussed here for the sake of space restrictions). However, we have found only two precedents of the interactions of macrolides and macrolactams with their receptors: the docking of dictyostatin into the taxol binding site [34] by Glide, AutoDock 4.0 and RosettaLigand [35]; and that of spinosyn precursors (22-membered macrolides) and their products by AD Vina and DOCK [36].

**Figure 1:**
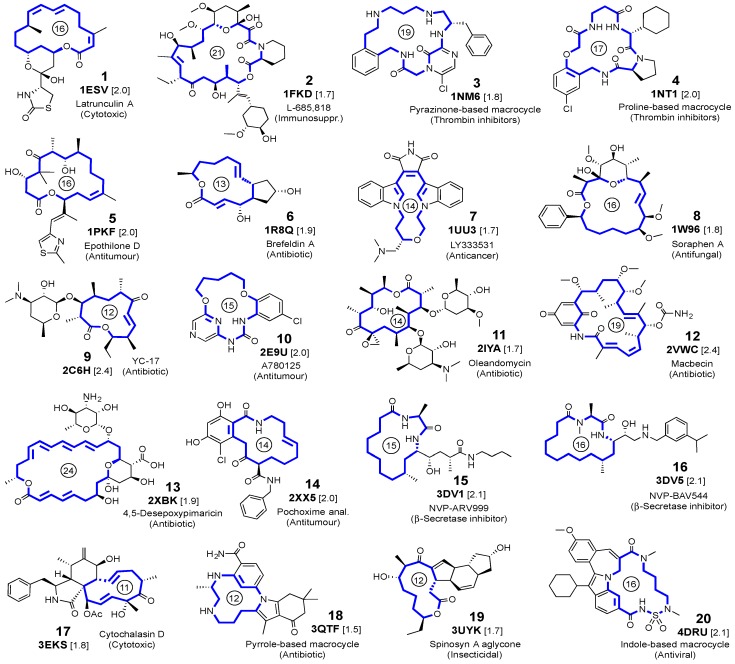
Selected ligands, with the PDB code and resolution (in Å, within brackets) of the crystalline complexes. For each macrocyclic ligand, the ring size is indicated by a number inside a circle.

Our objective was to evaluate how reliably these programs reproduce a set of protein–macrolide complexes for which crystallographic structures were reported with a good resolution (there are only eight disparate representative examples to date). Since this number is low, in our set we included some macrolactams, cyclodepsipeptides, and other heteroatom-containing macrocycles of similar size to our desired macrolides for which high-resolution crystallographic structures were also published. This gave us a total of 20 examples, which we ordered according to their PDB code (Figure 1). Six of these complexes (**1NTI**, **1UU3**, **1W96**, **2IYA**, **2XBK**, and **3EKS**) have just been examined using other methods [37]. We envisage expanding our test to other programs in the future and applying the most efficient to dock macrolide-like anti-tumor agents synthesized by our research group into cytoskeleton proteins.

## 2. Results and Discussion

### 2.1. Self-Docking of the Twenty PDB Structures

First of all, a self-docking study (cognate docking) was undertaken in which each ligand of Figure 1 was separated from each receptor (protein) and each pair was then subjected to a rigid docking calculation. We first used the standard parameters given for each program.

The results obtained with the five programs mentioned in the introduction are shown in Table 1. All the methods gave very good results. In fact, the root mean square deviation values were quite low (mean RMSD ≤ 1.0 Å); the standard errors of these mean RMSDs (SEM) are also indicated. The standard deviations (SD) are shown in the penultimate row and the median values in the last row. There are other appropriate statistical methods to evaluate the self-docking performance [38], but the mean and median RMSD values are sufficiently representative.

**Figure 2:**
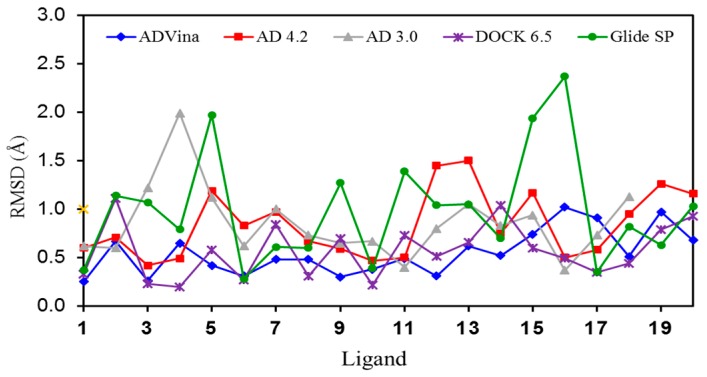
Graphic representation of the RMSD values for the self-docking of ligands **1**–**20** into their corresponding binding sites (crystal structures).

The lowest mean RMSD values were those provided by AD Vina (0.55) and DOCK (0.57). The speed of AD Vina was worth noting (just a few seconds per calculation), whereas the other programs required ca. 5 min per calculation. Figure 2 shows graphically which complexes were better or more poorly reproduced in silico.

When only the macrolide subset (**1**, **5**, **6**, **8**, **9**, **11**, **13**, and **19**) was taken into account, the average RMSD values turned out slightly better than the averages for all 20 compounds shown in Table 1:

AD Vina, 0.48 < DOCK, 0.55 < AD 3.0, 0.82 < AD 4.2, 0.89 < Glide, 0.94

### 2.2. Ligand Conformational Search

The database of conformers for each ligand was obtained by means of the MD/LLMod method. The values of the RMSD between the crystal and the predicted structures are widely used to confirm whether a close-match docked pose was predicted or not by the docking simulation. An RMSD value ≤ 2 Å is fairly good.

Conformers lying 0–42 kJ·mol^−1^ (ca. 0–10 kcal/mol) above the lowest-energy minimum were retrieved. For some structures (e.g., for **17/3EKS**) a set of as few as 17 conformers was obtained, whereas for larger macrocycles >250 conformations had to be calculated (see Table 2). For the cases **15/3DV1** and **16/3DV5**, with more rotatable bonds, the numbers of conformers were 1106 and 1402, respectively.

### 2.3. Docking of all the Conformers of Each Ligand

**Figure 3:**
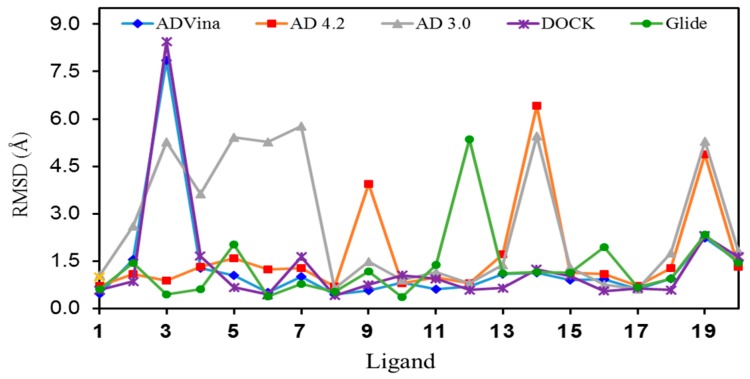
Graphic representation of the RMSD values for the lowest energy pose of each ligand (**1**–**20**) in their binding sites.

The RMSD values obtained for the lowest-energy poses predicted for each ligand are shown in Table 2 and the corresponding graphic representation is shown in Figure 3. The mean RMSD values for Glide, AD Vina, and DOCK were as low as 1.29/1.31/1.34 Å, respectively. The mean RMSD value for AD 4.2 turned out to be slightly higher (1.69 Å), while for AD 3.0 was 2.62 Å.

From a statistics viewpoint, we have also included at the bottom of Table 2 the mean RMSDs after ruling out the 2–4 RMSD values drawn in red (outliers) in the corresponding columns. It is clear that all the methods, except AD 3.0, afford very similar results. The RMSD values for each conformer and a Table with statistical results are given as Supplementary Material.

As mentioned above, the five docking programs were initially examined using their standard parameters. However, “flexible rings docking” with the program AD 4.2 (“AD 4.2 Flex”), which allows for the flexibility of the macrocycle, was also carried out. Since poorer values were obtained than those under the standard conditions, this option was not considered further. No induced fit approaches, taking into account the possible flexibility of the side chains of the receptors, were checked for any program, since we did not perform virtual screenings, but instead evaluated the capacity of the different docking programs to reproduce a set of complexes that contain poly-functional ligands. The ligands were relatively large, compared to most “small molecules” (drugs), and they contained a relatively rigid core (the macrocycle).

The case of **3/1NM6** requires some comments. All the methods, with the exception of AD 4.2 and Glide, gave RMSD values that were too high. A look at the docking images indicated that there are similar hydrophobic moieties (aromatic rings) at both extremes of **3** and some docking programs indistinctly located the folded conformers in either arrangement; they could not distinguish one pose from the other.

The fact is that, for each program, there were 2–4 complexes in which the ligands had too high RMSD values. If these two “worst points” were removed, the mean RMSD values decreased significantly. For instance, the mean RMSD values for AD Vina, DOCK, and Glide decreased to 0.90, 0.94, and 0.95, respectively. In other words, 80%–90% of the structures seemed to be well reproduced.

As far as the calculation speed is concerned, some representative computing times with fourth-generation i7 processors are indicated. With **5/1PKF** (412 conformers) the order was:

Glide, 5 min < AD Vina, 1 h < DOCK, 2 h < AD 4.2, 19 h < AD 3.0, 26 h

In the case of **16/3DV5** (1402 conformers), the order was:

AD Vina, 5 h < Glide, 10 h < AD 4.2, 39 h < AD 3.0, 44 h < DOCK, 52 h

Thus, AD Vina is not only among the three most accurate methods but it is quite rapid as well. AD 4.2 appears in an intermediate position (good accuracy but slow), while AD 3.0 offered the worst results while requiring more computer time than any other (the least efficient). This is understandable since AD 3.0 is an old version of AD 4.2. We needed to evaluate the difference, since, as commented in the introduction, we had often used the program more than a decade ago.

DOCK turned out to be as accurate as AD Vina, as happened in the self-docking procedure, but it was slower than AD Vina. For molecules with a very large number of conformers, the time increased exponentially. The performance of Glide, taking into account that the overall time invested was usually very low, was excellent. However, for molecules with a very large number of conformers, the time grew exponentially, as happened with DOCK. All the calculations with Glide were carried out with the standard precision (SP) method, but see the next section for the use of Glide XP.

When only the macrolide subset (**1**, **5**, **6**, **8**, **9**, **11**, **13**, and **19**) was analyzed, the average RMSD values were slightly better than the averages for all 20 compounds shown in Table 2:

DOCK, 0.84 < AD Vina, 0.87 < Glide, 1.20 < AD 4.2, 1.98 < AD 3.0, 2.71

### 2.4. Comparison of AD Vina with PSOVina

With the macrolide subset, we estimated how much the new algorithm contained in PSOVina [31] reduced the calculation time. As shown in Table 3, in six out of the eight instances there were time saving, operating with the same computer, equipped with an i7-4790 processor.

The RMSD values were similar, except for the complex of ligand **11/2IYA**, for which it was higher with PSOVina (0.96) than with AD Vina (0.62). Owing only to this difference, the average RMSD value for PSOVina turned out to be slightly higher. Notwithstanding, the predicted binding affinities were practically identical.

### 2.5. Poses with Lower RMSD Values

In several cases, the lowest-energy conformer predicted for each ligand was not the best pose (lowest RMSD). There was another conformer with a lower RMSD, the binding affinity of which was only around 0.0–0.5 kcal/mol above that of the lowest energy. In other words, there was often a conformer that overlapped with the crystalline structure better—we might call them the active conformer or that closest to the bioactive form, assuming that in many cases, but not always, the conformation of the ligand in the crystal structure may be the active conformation—while the score was only slightly different from that reported in Table 2. A summary of the results is shown in Table 4. At the bottom of Table 4, the average RMSDs together with the standard deviations are indicated for each method. Thus, for AD Vina the RMSD values are 0.83 ± 0.39 (they fall between 0.44 and 1.22), for AD 4.2 between 0.63 and 1.31, for DOCK between 0.45 and 1.41, and for Glide between 0.38 and 1.46. The medians are also included. It is observed that the mean and median RMSD values obtained with AD 3.0 are much higher than the rest. In short, all the methods (except AD 3.0) give nearly similar outcomes.

Table 4 also shows the rank or pose order of the conformer with the optimal binding affinity; the number of red numbers in the pose-order columns is diagnostic (AD 3.0 is the worst in this sense).

A comparison of Table 2 and Table 4 indicates that the performance of all the methods significantly improved, with the exception of two cases: (a) for **12/2VWC**, Glide predicted an unacceptable RMSD value of 5.36; (b) for **14/2XX5**, AD 3.0 gave an RMSD value of 4.81.

Again, when only the macrolide subset (**1**, **5**, **6**, **8**, **9**, **11**, **13**, and **19**) was considered, the average RMSD values changed slightly in relation to the general averages shown in Table 4:

AD Vina ≈ DOCK, 0.75 < Glide, 1.00 < AD 4.2, 1.05 < AD 3.0, 1.08

### 2.6. Re-Scoring

Re-docking of the main poses with computational methods that provide binding affinities closer to experimental values has a long tradition. Among the successful re-scorings reported, we will mention a few that have been published very recently [39,40,41,42,43,44,45,46]. We realized that although AD Vina is a good compromise between speed and accuracy, we observed that the gaps between the predicted binding affinities for the best pose and for the next poses are minimal. In fact, many poses had very close scoring functions (often with differences of only 0.1 kal/mol).

**Figure 4:**
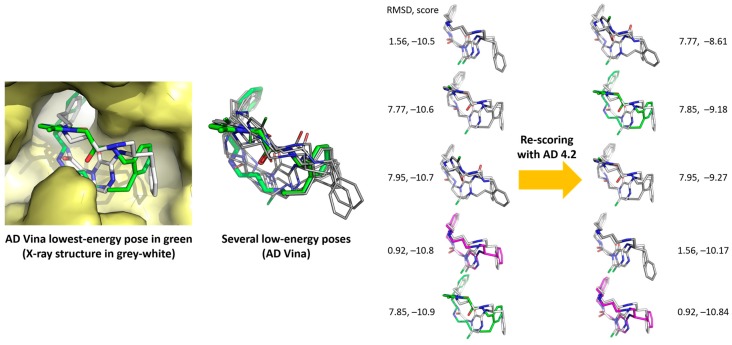
Re-scoring with AD 4.2 (right column) of AD Vina-predicted main poses of **3/1NM6**.

Re-scoring of the 20 molecules with X-Score software [47] did not increase the small gaps. Re-scoring the AD Vina poses with AD 4.2, which obviously has advantages regarding the data compatibility between programs, improved the gaps between the poses, as shown in Figure 4 for the case of **3/1NM6**. It should be noted that many conformations lying between −10.9 and −10.5 kcal/mol, only a few of which are shown in Figure 4 for the sake of simplicity, gave rise to larger energy differences after re-scoring: from −10.84 to −8.61 kcal/mol, in the examples shown. The conformer depicted in green (that of the lowest energy, −10.9 kcal/mol, according to AD Vina, see Table 2) moved up to −9.18 kcal/mol. The conformer depicted in magenta, which was not the lowest-energy one according to AD Vina (but it was the best pose), became the most favorable after the re-scoring.

We call this re-scoring AutoDock 4.2//AutoDock Vina, or just AD 4.2//Vina, as done in quantum chemistry from the very beginning for single-point calculations of the energy at a high level of theory on equilibrium geometries obtained at a lower level. Table 5 summarizes the results for the AD 4.2//Vina re-scoring of the full series.

Comparison with preceding Tables indicates how reliable this approach is. The mean RMSD and standard deviation does not significantly change (from 0.97 ± 0.34 to 1.05 ± 0.39), whereas the pose order of the lowest-energy predicted conformer moves to one (with only one exception, see **14**). In other words, with AD 4.2//Vina, all the best poses for each ligand except one also appeared to be the lowest-energy conformers, in sharp contrast with the results in Table 4 for AD 4.2 (repeated in Table 5 for the sake of a direct comparison) and AD Vina. This may seem a fortuitous fact, to be interpreted by the authors of AutoDock, but it was well known that the scoring functions of AD 4.2 are more reliable than those of AD Vina, while AD Vina runs more rapidly than AD 4.2. Although the average RMSD value increased slightly (see Table 5), it is lower than the average values of 1.31 and 1.69 in Table 2 for AD Vina and AD 4.2, respectively.

Taking the 20 poses of lowest energy in each case, we also tested the re-scoring of the four poorest DOCK results in Table 4 with GBSA score, which uses the pairwise GB solvation model [48] with special parameters [49], and with Amber Score [50], which includes an implicit solvation and a short MD simulation that takes into account some flexibility of the receptor. Whereas no improvement was observed for **3/1NM6**, poses were found for complexes **7/1UU3**, **15/3DV1**, and **19/3UYK** which showed the optimal RMSD values and optimal relative energies, especially with the Amber Score (see Table 6).

We also performed Glide XP calculations (eXtra Precision, as a matter of fact a docking procedure with more precise scoring functions) [51] and a re-scoring with the popular PRIME MM-GBSA module [52] for the six poorest results in Table 4, which were clearly improved with both programs in all cases (Table 6).

Finally, we examined whether these poses, which are in principle more reliable, overlap with the experimental pose (crystalline structure) or not. The drawings and RMSD for the superimpositions (RMSD overlap) are given as Supplementary Material, but the key points are:

(a) With AD 4.2//Vina, 9 out of the 20 ligands overlap almost perfectly with their respective poses in the crystals (ligands **1**, **3**, **7**, **8**, **9**, **10**, **12**, **17**, and **18**, RMSD overlap = 0.32–0.67 Å), 6 ligands can be superimposed moderately well with their crystalline structures, at least their cyclic moieties (RMSD overlap = 0.79–0.93), and there are four calculated structures that do not match sufficiently well (**5**, **11**, **13,** and **14**, RMSD overlap = 1.04–1.10). The worst result is for ligand **19** (RMSD overlap = 2.14); we have no explanation for it, but the crystalline structure of the complex was poorly reproduced by most methods (see Table 2, Table 3, Table 4, Table 5 and Table 6).
(b) Three poses that improved after re-scoring of DOCK with Amber (Table 6) showed values of RMSD overlap for **7**, **15**, and **19** equal to 0.87, 1.12, and 0.75, respectively (two match partially and one not sufficiently well, also by visual inspection).
(c) For the six Glide poses re-scored with MM-GBSA (Table 6), the RMSD-overlap values were 1.13, 1.36, 0.69, 0.64, 0.73, and 0.67. In other words, three ligands (**12**, **13**, and **19**) were almost superimposable with their respective experimental structures, but there were two that did not match well. It is worth noting that **19/3UYK** is better described by Glide (docking and re-scoring) than by AD 4.2 (docking and AD 4.2//Vina re-scoring, respectively).

## 3. Materials and Methods

### 3.1. Crystalline Complexes

The proteins selected for docking analysis satisfied the following criteria: they do not form covalent bonds with their respective ligands; the main rings have ≥11 members; and the structures were refined at resolutions <2.5 Å (Figure 1).

### 3.2. Preparation of the Ligand Files

Ligand coordinate files were extracted from the corresponding PDB files and used as reference structures for root mean square deviation (RMSD) calculations. The ligand input files were represented from their one-dimensional notations, SMILEs, with the aim of avoiding any bias from the bioactive conformation in conformer calculations. The 3D structures were generated with Corina [53] and were refined with LigPrep [54].

### 3.3. Conformer Calculations

Macrocycle from the MacroModel 10.0 package was used for the conformational searches [55]. OPLS2005 was the force field selected. The solvent effect was described by means of the GB/SA continuum solvent model. From the different methods of conformational search, we chose molecular dynamics/large-scale low-mode sampling (MD/LLMod) [56,57]. This MD/LLMod method [56] is based on a two-stage search strategy. The first stage is high temperature MD-based simulated annealing, while the second stage is a large-scale low-mode search (LLMod). The MD/LLMod parameters were obtained after 5000 simulated annealing cycles and then 5000 energy-minimization iterations, with a maximum of 42 kJ·mol^−1^ (ca. 10 kcal/mol) above the lowest-energy conformer.

### 3.4. Preparation of the Protein Files

The receptor input files were prepared as usual. First, we removed all water molecules, ligand atoms, and those ions that do not belong to the active site of the receptor from the corresponding PDB file. Next, hydrogen atoms were added, as well as protons, and amino acid side-chain partial charges (in accordance with the protonation state at the physiological pH) were assigned according to the requirements of each program. This was followed by a local minimization to relieve potential bad contacts. The minimization was performed in the presence of restraints to maintain the protein conformation very close to that observed in the experimental model. For those proteins with one cytochrome molecule close to the binding site (**1PKF**, **2C6H**, and **2XBK**), the Chimera USCF program [58] was used; the corresponding hydrogen atoms and the AM1-Bcc partial charges of each atom were added and the cytochrome molecule was then included.

### 3.5. Docking Algorithms

AutoDock programs use a stochastic search algorithm (Lamarckian Genetic Algorithm, LGA, for AutoDock 3.0 and 4.2.6, and Iterated Local Search, ILS, for AutoDock Vina and PSO Vina) [28,29,30,31]. DOCK and Glide use a systematic search algorithm [32,33].

### 3.6. Calculations of RMSD Values

Docking results were assessed with RMSD (root-mean-square deviation of the heavy atoms) of each predicted pose versus the crystal structure. The RMSD values were calculated with the rmsd.py script downloaded from the Script Center of the Schrödinger Resources website.

## 4. Conclusions

We evaluated the performance of some popular docking programs at reproducing X-ray crystallographic structures that contain macrolides and related poly-functionalized ligands. Thus, the conclusions that follow regarding the performance of each method refer only and exclusively to these macrocyclic substrates when bound to rigid receptors; any generalization to other types of complexes will or would require an independent study. To our initial surprise, the outcome was very satisfactory, as most of the programs arrived at optimal poses in spite of the complexity of the structures. In general, for our set of 20 complexes, Glide 6.6 (Glide), AutoDock Vina (AD Vina), and DOCK 6.5 (DOCK) were efficient docking procedures for macrolides and analogues of intermediate size (Table 2). For large structures with many conformational isomers, the computing times of the programs Glide and especially of DOCK grew exponentially. If only true macrolides are considered (eight examples available), the preceding sentences are still valid. AD 4.2 outperforms AD 3.0, as expected for an updated and improved version, but we needed to confirm that this was the case for macrolide-like ligands. Consequently, our results obtained with AD 3.0 more than 10 years ago should be revised and corrected. When the best poses were compared (Table 4), AD Vina, Glide, DOCK, and AD 4.2 gave mean RMSD values below 1.0 Å. Since the energies given by AD Vina for the different conformers are very close, re-scoring with AD 4.2 (here so-called AD 4.2//Vina) was performed. This increased the energy gaps between the different poses and the agreement between the computational results and crystalline structures significantly improved. In general, re-scorings of DOCK and Glide also improved the agreement, which could have been expected but it had to be demonstrated for macrolide-like compounds. We hope to extend this study to other docking programs and to take advantage of the present findings in connection with the design and synthesis of analogs of anti-tumor macrolides.

## Figures and Tables

**Table 1:** RMSD values (in Å) and scoring energies ^a^ for the self-docking of each ligand in its binding site.

		AD Vina		AD 4.2		AD 3.0		DOCK		Glide	
		RMSD	Score	RMSD	Score	RMSD	Score	RMSD	Score	RMSD	Score
**1**	**1ESV**	0.25	−10.8	0.60	−8.52	0.62	−10.40	0.33	−67.42	0.37	−7.84
**2**	**1FKD**	0.67	−11.3	0.71	−11.10	0.66	−12.38	1.11	−61.33	1.14	−6.95
**3**	**1NM6**	0.26	−12.8	0.42	−12.79	1.22	−12.24	0.23	−88.46	1.07	−11.32
**4**	**1NT1**	0.65	−13.3	0.49	−13.50	1.99	−6.89	0.20	−85.14	0.79	−9.68
**5**	**1PKF**	0.42	−13.7	1.19	−12.73	1.12	−13.67	0.58	−77.96	1.97	−8.32
**6**	**1R8Q**	0.31	−12.6	0.83	−10.66	0.62	−11.69	0.27	−65.97	0.28	−11.73
**7**	**1UU3**	0.48	−11.9	0.97	−11.69	1.00	−12.88	0.84	−84.54	0.61	−10.27
**8**	**1W96**	0.48	−11.7	0.67	−12.03	0.73	−13.92	0.31	−95.40	0.60	−8.86
**9**	**2C6H**	0.30	−11.1	0.59	−9.99	0.65	−12.36	0.70	−79.95	1.27	−6.61
**10**	**2E9U**	0.38	−10.2	0.47	−9.10	0.67	−10.20	0.22	−67.52	0.39	−8.68
**11**	**2IYA**	0.49	−13.2	0.50	−11.60	0.40	−14.73	0.73	−84.69	1.39	−9.37
**12**	**2VWC**	0.31	−9.4	1.45	−9.15	0.80	−10.43	0.51	−78.13	1.04	−6.94
**13**	**2XBK**	0.62	−18.5	1.50	−15.23	1.05	−18.33	0.66	−115.34	1.05	−11.64
**14**	**2XX5**	0.52	−11.3	0.75	−10.58	0.83	−8.92	1.04	−73.64	0.70	−8.84
**15**	**3DV1**	0.74	−10.9	1.17	−11.57	0.94	−14.29	0.60	−94.39	1.94	−9.25
**16**	**3DV5**	1.02	−11.6	0.50	−15.36	0.37	−17.22	0.49	−113.99	2.37 ^b^	−8.20
**17**	**3EKS**	0.91	−11.5	0.58	−11.77	0.73	−12.06	0.35	−75.36	0.35	−10.00
**18**	**3QTF**	0.51	−13.3	0.95	−10.88	1.13	−12.57	0.44	−78.45	0.82	−11.53
**19**	**3UYK**	0.97	−10.7	1.26	−10.03	1.39	−10.76	0.79	−55.11	0.63	−7.92
**20**	**4DRU**	0.68	−13.1	1.16	−12.99	1.16	−12.92	0.93	−71.69	1.03	−8.78
mean		0.55 ± 0.05		0.84 ± 0.08		0.90 ± 0.08		0.57 ± 0.06		0.99 ± 0.13	
SD		0.23		0.34		0.37		0.28		0.57	
median		0.50		0.73		0.82		0.55		0.93	

^a^ Energies in kcal/mol, except for DOCK (in kJ·mol^−1^), as given by each method; ^b^ RMSD values higher than 2.00 are written in red throughout the Tables.

**Table 2:** RMSD values (in Å) ^a^ and scoring energies of the lowest-energy poses of **1**–**20**.

		No. Conf.	AD Vina		AD 4.2		AD 3.0		DOCK		Glide	
			RMSD	Score	RMSD	Score	RMSD	Score	RMSD	Score	RMSD	Score
**1**	**1ESV**	73	0.47	−10.7	0.72	−9.47	1.04	−11.56	0.59	−61.98	0.62	−8.44
**2**	**1FKD**	315	1.55	−9.1	1.09	−10.29	2.62	−9.89	0.86	−71.40	1.45	−7.00
**3**	**1NM6**	531	**7.85**	−10.9	0.89	−12.17	**5.28**	−10.67	**8.44**	−71.02	0.44	−10.20
**4**	**1NT1**	246	1.28	−13.2	1.33	−12.53	**3.63**	−11.50	1.66	−67.53	0.61	−11.43
**5**	**1PKF**	412	1.05	−13.3	1.59	−13.56	**5.41**	−14.72	0.67	−78.59	2.03	−8.86
**6**	**1R8Q**	109	0.50	−11.5	1.23	−10.43	**5.28**	−11.92	0.44	−63.66	0.38	−**11**.66
**7**	**1UU3**	38	1.00	−10.9	1.27	−11.16	**5.77**	−11.40	1.64	−83.79	0.78	−10.58
**8**	**1W96**	278	0.45	−11.7	0.69	−12.27	0.64	−12.66	0.41	−86.84	0.52	−8.92
**9**	**2C6H**	69	0.58	−10.9	**3.94**	−9.86	1.49	−11.83	0.75	−84.40	1.17	−7.02
**10**	**2E9U**	36	0.82	−10.9	0.79	−8.98	0.91	−10.12	1.06	−60.40	0.36	−8.75
**11**	**2IYA**	265	0.62	−11.8	0.98	−16.08	1.15	−14.82	0.94	−99.18	1.39	−9.99
**12**	**2VWC**	60	0.70	−9.8	0.79	−9.36	0.79	−10.87	0.59	−76.21	**5.36**	−6.15
**13**	**2XBK**	373	1.09	−16.3	1.72	−19.73	1.39	−18.66	0.65	−111.52	1.12	−12.84
**14**	**2XX5**	422	1.14	−11.3	**6.41**	−11.49	**5.47**	−9.68	1.24	−73.53	1.15	−8.96
**15**	**3DV1**	1106	0.90	−11.4	1.14	−13.05	1.29	−13.48	1.03	−96.73	1.14	−10.68
**16**	**3DV5**	1402	0.92	−12.4	1.10	−14.39	0.76	−16.47	0.56	−102.58	1.94	−8.04
**17**	**3EKS**	17	0.61	−10.8	0.71	−12.97	0.61	−12.37	0.64	−70.42	0.67	−10.02
**18**	**3QTF**	24	0.94	−13.4	1.28	−11.59	1.75	−13.73	0.59	−74.86	0.94	−11.62
**19**	**3UYK**	78	2.23	−10.0	**4.88**	−10.57	**5.30**	−10.78	2.30	−52.57	2.33	−7.46
**20**	**4DRU**	50	1.50	−13.1	1.31	−12.56	1.82	−12.90	1.64	−77.54	1.44	−8.23
mean			1.31 ± 0.36		1.69 ± 0.34		2.62 ± 0.45		1.34 ± 0.39		1.29 ± 0.25	
SD			1.60		1.54		2.01		1.75		1.11	
median			0.93		1.19		1.62		0.81		1.13	
mean corr. ^b^			0.90 ± 0.08		1.09 ± 0.07				0.94 ± 0.10		0.95 ± 0.11	

^a^ Energies in kcal/mol, except for DOCK (in kJ·mol^−1^), as given by each method. The highest (poorest) values are indicated in bold red. Poor RMSD values (above 2.0 Å but below 2.5 Å) written in red; ^b^ Mean values and standard deviations ruling out the 2–4 RMSD values drawn in red (outliers).

**Table 3:** Comparison of AD Vina and PSOVina results for the lowest-energy conformers of the macrolide subset.

		No. of Conform.	AD Vina			PSOVina		
			RMSD	Score	Time	RMSD	Score	Time
**1**	**1ESV**	73	0.47	−10.7	8 min	0.47	−10.71	5 min
**5**	**1PKF**	412	1.05	−13.3	71 min	1.04	−13.26	52 min
**6**	**1R8Q**	109	0.50	−11.5	11 min	0.48	−11.50	8 min
**8**	**1W96**	278	0.45	−11.7	66 min	0.45	−11.72	50 min
**9**	**2C6H**	69	0.58	−10.9	11 min	0.58	−10.85	15 min
**11**	**2IYA**	265	0.62	−11.8	180 min	0.96	−11.86	85 min
**13**	**2XBK**	373	1.09	−16.3	225 min	1.10	−16.24	101 min
**19**	**3UYK**	78	2.23	−10.0	6 min	2.25	−10.01	10 min
mean			0.87 ± 0.21			0.92 ± 0.21		
SD			0.60			0.60		
median			0.60			0.77		

**Table 4:** RMSD values (in Å) and scoring energies for the best poses (the lowest RMSDs) of **1**–**20**.

		No. Conf.	AD Vina			AD 4.2			AD 3.0			DOCK			Glide		
			RMSD	Score	Pose ^a^	RMSD	Score	Pose	RMSD	Score	Pose	RMSD	Score	Pose	RMSD	Score	Pose
**1**	**1ESV**	73	0.47	−10.7	1	0.61	−9.29	**5**	0.82	−11.24	4	0.59	−61.98	1	0.36	−8.31	3
**2**	**1FKD**	315	1.04	−9.0	2	1.09	−10.29	1	1.63	−9.12	**5**	0.86	−71.40	1	1.12	−6.94	**6**
**3**	**1NM6**	531	0.92	−10.8	3	0.89	−12.17	1	2.34	−10.10	3	**2.31**	−67.63	**7**	0.44	−10.20	1
**4**	**1NT1**	246	1.28	−13.2	1	0.85	−11.63	**9**	2.90	−11.18	4	1.66	−67.53	1	0.48	−11.14	2
**5**	**1PKF**	412	0.58	−12.8	**9**	1.20	−13.05	**8**	2.07	−14.18	**8**	0.67	−78.59	1	2.03	−8.86	1
**6**	**1R8Q**	109	0.50	−11.5	1	0.84	−10.31	3	0.75	−11.52	**7**	0.44	−63.66	1	0.38	−11.66	1
**7**	**1UU3**	38	1.00	−10.9	1	1.27	−11.16	1	1.00	−11.07	**5**	1.14	−79.11	4	0.78	−10.58	1
**8**	**1W96**	278	0.45	−11.7	1	0.69	−12.27	1	0.64	−12.66	1	0.41	−86.84	1	0.52	−8.92	1
**9**	**2C6H**	69	0.56	−10.8	2	0.79	−9.69	**5**	0.88	−11.46	**5**	0.75	−84.40	1	0.82	−6.85	**15**
**10**	**2E9U**	36	0.82	−10.9	1	0.79	−8.98	1	0.91	−10.12	1	1.06	−60.40	1	0.36	−8.75	1
**11**	**2IYA**	265	0.52	−11.7	**5**	0.98	−16.08	1	0.53	−14.64	4	0.94	−99.18	1	1.39	−9.99	1
**12**	**2VWC**	60	0.70	−9.8	1	0.79	−9.36	1	0.79	−10.87	1	0.59	−76.21	1	**5.36** ^c^	−6.15	1
**13**	**2XBK**	373	0.66	−16.2	3	1.72	−19.73	1	1.39	−18.66	1	0.65	−111.52	1	0.80	−12.74	4
**14**	**2XX5**	422	0.77	−10.8	**5**	1.74	−10.42 ^b^	**43**	**4.81**	−9.59	**8**	1.24	−73.53	1	0.91	−8.91	4
**15**	**3DV1**	1106	0.90	−11.4	1	0.92	−12.80	**6**	1.29	−13.48	1	0.86	−95.70	**8**	1.14	−10.68	1
**16**	**3DV5**	1402	0.92	−12.4	1	0.67	−14.14	**8**	0.76	−16.47	1	0.56	−102.58	1	1.94	−8.04	1
**17**	**3EKS**	17	0.61	−10.8	1	0.71	−12.97	1	0.61	−12.37	1	0.45	−69.80	4	0.31	−10.00	2
**18**	**3QTF**	24	0.56	−13.3	2	0.81	−11.14	**9**	1.75	−13.73	1	0.59	−74.86	1	0.52	−10.97	3
**19**	**3UYK**	78	**2.23**	−10.0	1	1.56	−9.77	**10**	1.57	−10.24	**5**	1.57	−49.93	**15**	1.69	−7.45	2
**20**	**4DRU**	50	1.07	−12.9	**5**	1.31	−12.56	1	1.82	−12.90	1	1.30	−76.28	3	1.44	−8.23	1
mean			0.83 ± 0.09			0.97 ± 0.08			1.46 ± 0.22			0.93 ± 0.11			0.92 ± 0.25		
SD			0.39			0.34			0.99			0.48			0.54		
median			0.74			0.87			1.15			0.81			0.81		

^a^ Pose order (1 = the lowest-energy pose). When the order number is 2, 3, or 4 it is indicated in red; when the order number is ≥5 in bold red; ^b^ This is an exception: it is 1.0 kcal/mol above that of the lowest-energy conformer (instead of 0.0–0.5 kcal/mol), but poses 1–42 showed higher RMSDs; ^c^ No conformation with lower RMSD was found with a gap lower than 1.5 kcal/mol (better RMSD = 3.15, −4.84 kcal/mol, pose order = 36).

**Table 5:** Re-scoring. AD 4.2//Vina results.

		AD 4.2 (Table 4)			AD 4.2//Vina		
		RMSD	Score	Pose	RMSD	Score	Pose
**1**	**1ESV**	0.61	−9.29	**5**	0.47	−8.68	1
**2**	**1FKD**	1.09	−10.29	1	1.20	−8.79	1
**3**	**1NM6**	0.89	−12.17	1	0.92	−10.84	1
**4**	**1NT1**	0.85	−11.63	**9**	1.28	−12.20	1
**5**	**1PKF**	1.20	−13.05	**8**	1.23	−12.66	1
**6**	**1R8Q**	0.84	−10.31	3	0.98	−9.90	1
**7**	**1UU3**	1.27	−11.16	1	1.00	−11.51	1
**8**	**1W96**	0.69	−12.27	1	0.45	−12.07	1
**9**	**2C6H**	0.79	−9.69	**5**	0.58	−10.15	1
**10**	**2E9U**	0.79	−8.98	1	0.82	−8.70	1
**11**	**2IYA**	0.98	−16.08	1	1.23	−14.99	1
**12**	**2VWC**	0.79	−9.36	1	0.70	−9.27	1
**13**	**2XBK**	1.72	−19.73	1	1.19	−17.10	1
**14**	**2XX5**	1.74	−10.42	**43**	1.59	−10.52	**4**
**15**	**3DV1**	0.92	−12.80	**6**	0.97	−12.45	1
**16**	**3DV5**	0.67	−14.14	**8**	1.05	−13.92	1
**17**	**3EKS**	0.71	−12.97	1	1.14	−12.44	1
**18**	**3QTF**	0.81	−11.14	**9**	0.98	−11.99	1
**19**	**3UYK**	1.56	−9.77	**10**	2.23	−9.36	1
**20**	**4DRU**	1.31	−12.56	1	1.07	−13.20	1
mean		0.97 ± 0.08			1.05 ± 0.09		
SD		0.34			0.39		
median		0.87			1.03		

**Table 6:** Re-scoring of DOCK and Glide.

		**DOCK 6.5**			
		**Re-Scoring**		**Re-Scoring**	
		**RMSD**	**GBSA**	**RMSD**	**Amber**
**7**	**1UU3**	1.69	−77.37	1.10	−58.20
**15**	**3DV1**	1.44	−70.39	1.42	−40.49
**19**	**3UYK**	2.30	−43.23	1.39	−41.89
		**Glide**			
		**Re-Scoring**		**Re-Scoring**	
		**RMSD**	**XP**	**RMSD**	**MM-GBSA**
**2**	**1FKD**	1.35	−9.15	1.47	−108.69
**9**	**2C6H**	1.11	−7.00	1.49	−113.55
**12**	**2VWC**	0.95	−6.80	0.95	−91.98
**13**	**2XBK**	1.04	−14.54	0.83	−153.96
**14**	**2XX5**	1.10	−10.13	1.10	−97.36
**19**	**3UYK**	1.93	−12.30	1.71	−90.04

## Supplementary Materials

The following are available online at http://www.mdpi.com/1420-3049/22/1/136/s1. Table S1: Features of the crystal structures downloaded from the Protein Data Bank. Table S2: Properties of the ligands. Table S3: Conformational statistics. Figure S1: Ligand **9** with the density map downloaded and as it appears in the chemical literature. Figure S2: Structures of the docked ligands (AD 4.2//Vina). Figure S3: Docked ligands (Amber from DOCK). Figure S4: Docked ligands (MM-GBSA from Glide). Table S4: Comparison of AD 4.2 with AD 4.2 Flex. Figure S5: Structures of **7**, **16**, and **19** (AD 4.2 Flex vs. AD 4.2).
