# Supplementary material for: The Performance of Several Docking Programs at Reproducing Protein–Macrolide-Like Crystal Structures
Source: Molecules. 2017 Jan 17;22(1):136. doi: 10.3390/molecules22010136 (PMC6155922; doi:10.3390/molecules22010136)
Supplement: Supplementary file 1 [file molecules-22-00136-s001.pdf]

Supplementary Materials: The Performance of Several Docking Programs at Reproducing Protein–Macrolide-Like Crystal Structures

Alejandro Castro-Alvarez, Anna M. Costa and Jaume Vilarrasa

Table S1. Features of the crystal structures downloaded from the Protein Data Bank 2

Table S2. Properties of the ligands 3

1. RMSD values for each conformer 4

2. The C7 stereocenter of **9** (YC-17, complex 2C6H) 5

3. $RMSD_{overlap}$ 6

4. AutoDock 4.2 vs. AutoDock 4.2 Flex 8

Table S1. Features of the crystal structures downloaded from the Protein Data Bank.

Ligand	PDB ID	Resoln (Å)	Classification	Ring Size	No. Conform.	Ligand Name
**1**	1ESV	2.0	macrolide	16	73	Latrunculin A
**2**	1FKD	1.7	cyclodepsipeptide	21	315	18-Hydroxyascomycin
**3**	1NM6	1.8	hybr. macrolactam	19	531	(11*S*)-11-Benzyl-6-chloro-1,2,10,11,12,13,14,15,16,17,18,19-dodecahydro-5,9-methano-2,5,8,10, 13,17-benzohexaazacyclohenicosine-3, 24(4*H*)-dione
**4**	1NT1	2.0	hybr. cyclopeptide	17	246	(6*R*,21a*S*)-17-Chloro-6-cyclohexyl-2,3,6,7,10,11,19,20-octahydro-1*H*,5*H*-pyrrolo[1,2-*k*][1,4,8,11,14]benzoxatetraazacycloheptadecine-5,8,12,21(9*H*, 3*H*, 21a*H*)-tetrone
**5**	1PKF	2.0	macrolide	16	412	Epothilone D
**6**	1R8Q	1.9	macrolide	13	109	Brefeldin A
**7**	1UU3	1.7	polycycl. compd.	14	38	LY333531
**8**	1W96	1.8	macrolide	16	278	Soraphen A
**9**	2C6H	2.4	macrolide	12	79	YC-17
**10**	2E9U	2.0	hybr. cyclic urea	15	36	A780125
**11**	2IYA	1.7	macrolide	14	265	Oleandomycin
**12**	2VWC	2.4	hybr. macrolactam	19	60	Macbecin
**13**	2XBK	1.9	macrolide	24	373	4,5-Desepoxypimaricin
**14**	2XX5	2.0	macrolactam	14	422	(6*E*,11*R*)-15-Chloro-16,18-dihydroxy-2,12-dioxo-*N*-(phenylmethyl)-3-azabicyclo[12.4.0]octadeca-1(14),6,15,17-tetraene-11-carboxamide
**15**	3DV1	2.1	macrolactam	15	1106	NVP-ARV999
**16**	3DV5	2.1	macrolactam	16	1402	NVP-BAV544
**17**	3EKS	1.8	polycycl. compd.	11	17	Cytochalasin D
**18**	3QTF	1.5	polycyclic compd.	12	24	(6*S*)-6,15,15,18-Tetramethyl-17-oxo-2,3,4,5,6,7,14,15,16,17-decahydro-1*H*-8,12-(metheno)[1,4,9]-triazacyclotetradecino[9,8-*a*]indole-9-carboxamide
**19**	3UYK	1.7	polyc. macrolide	12	78	Spinosyn A aglycone
**20**	4DRU	2.1	macropolycycle	16	50	13-Cyclohexyl-3-methoxy-17,22-dimethyl-7*H*-10,6-(methanoiminothioiminobutanoiminomethano)-indolo[2,1-*a*][2]benzazepine-14,23-dione 16,16-dioxide

Table S2. Properties of the ligands.

Ligand	PDB ID	SMILES notation	Heavy Atoms	log *P*	MW	TPSA [a]	No. Ac-Ceptors [b]	No. Donors [c]
**1**	1ESV	C[C@H]1CC[C@@H]2C[C@H](C[C@@](O)(O2)[C@@H]2CSC(=O)N2)OC(=O)\C=C(C)/CC\C=C\C=C/1	29	3.52	421	84.865	6	2
**2**	1FKD	O1[C@H](/C(=C/[C@H]2C[C@@H](OC)[C@H](O)CC2)/C)[C@H](C)[C@@H](O)CC(=O)[C@@H](\C=C(/C)\[C@@H](O)[C@@H](C[C@H](OC)[C@H]2O[C@](O)([C@@H](C[C@@H]2OC)C)C(=O)C(=O)N2[C@@H](CCCC2)C1=O)C)CC	57	3.31	807	198.604	14	4
**3**	1NM6	Cl[C@H]1N2CC(=O)NCc3c(CCNCCCNC[C@@H](N[C@H](N=C1)C2=O)Cc1ccccc1)cccc3	36	1.66	509	97.852	8	4
**4**	1NT1	Clc1cc2CNC(=O)[C@H]3N(CCC3)C(=O)[C@H](NC(=O)CCNC(=O)COc2cc1)C1CCCCC1	35	1.84	504	116.837	9	3
**5**	1PKF	s1cc(nc1C)\C=C(/C)\[C@H]1OC(=O)C[C@H](O)C(C)(C)C(=O)[C@H](C)[C@@H](O)[C@H](CCC\C(=C/C1)\C)C	34	4.50	491	96.724	6	2
**6**	1R8Q	O1[C@H](CCC\C=C\[C@H]2[C@@H](C[C@@H](O)C2)[C@H](O)CCC1=O)C	20	1.80	280	66.761	4	2
**7**	1UU3	O1CCn2c3c(c(C4=C(c5c6c(n(CC[C@H]1CN(C)C)c5)cccc6)C(=O)NC4=O)c2)cccc3	35	3.74	465	72.273	7	1
**8**	1W96	O1[C@@H](CCCC[C@H](OC)[C@H](OC)\C=C\[C@@H]([C@@H]2O[C@](O)([C@H](C)C1=O)[C@H](OC)[C@@H](O)[C@@H]2C)C)c1ccccc1	37	2.50	520	103.697	8	2
**9**	2C6H	O1[C@H](CC)[C@@H](\C=C\C(=O)[C@H](C[C@H](C)[C@H](O[C@H]2O[C@H](C[C@@H](N(C)C)[C@@H]2O)C)[C@@H](C)C1=O)C)C	32	1.70	453	85.310	7	1
**10**	2E9U	Clc1cc2NC(=O)Nc3nc(OCCCCCOc2cc1)cnc3	24	3.87	348	85.377	7	2
**11**	2IYA	CO[C@H]1C[C@H](O[C@H]2[C@H](C)[C@@H](O[C@@H]3O[C@H](C)C[C@@H]([C@H]3O)N(C)C)[C@@H](C)C[C@]3(CO3)C(=O)[C@H](C)[C@@H](O)[C@@H](C)[C@@H](C)OC(=O)[C@@H]2C)O[C@@H](C)[C@@H]1O	46	2.60	688	166.000	13	3
**12**	2VWC	O=C/1NC2=CC(=O)C=C([C@H](OC)[C@H](C[C@H](OC)[C@H](OC)[C@H](\C=C(/C)\[C@H](OC(=O)N)[C@H](/C=C\C=C\1/C)C)C)C)C2=O	40	0.34	558	143.270	10	3
**13**	2XBK	C[C@H]1O[C@@H](O[C@@H]2C[C@@H]3O[C@@](O)(C[C@H](O)[C@H]3C(O)=O)C[C@@H](O)C\C=C\C=C\C(=O)O[C@H](C)C\C=C\C=C\C=C\C=C\2)[C@@H](O)[C@@H](N)[C@@H]1O	46	1.80	650	218.470	13	8
**14**	2XX5	Clc1c2c(C(=O)NCC\C=C\CCC[C@@H](C(=O)NCc3ccccc3)C(=O)C2)c(O)cc1O	33	3.52	470	115.723	7	4
**15**	3DV1	O=C1N[C@@H](C[C@@H](CCCCCCCC(=O)N[C@H]1C)C)[C@@H](O)C[C@H](C(=O)NCCCC)C	31	3.70	439	107.522	7	4
**16**	3DV5	O=C1N[C@@H](C[C@@H](CCCCCCCCC(=O)N(C)[C@H]1C)C)[C@H](O)CNCc1cc(ccc1)C(C)C	35	5.57	485	81.662	6	3
**17**	3EKS	O=C1[C@H](C\C=C\[C@@H]2[C@]3([C@@H]([C@H](C)C(C)[C@H]2O)[C@@H](NC3=O)Cc2ccccc2)[C@H](OC(=O)C)\C=C\[C@]1(O)C)C	37	3.05	506	112.930	7	3
**18**	3QTF	O=C1CC(Cc2n-3c(CCCNC[C@@H](Nc4cc-3ccc4C(=O)N)C)c(c12)C)(C)C	30	3.53	408	89.153	6	4
**19**	3UYK	O1[C@H](CCC[C@H](O)[C@@H](C)C(=O)C2[C@H]([C@H]3[C@H]([C@H]4[C@@H](C[C@@H](O)C4)C=C3)C2)CC1=O)CC	29	2.71	402	83.832	5	2
**20**	4DRU	S1(=O)(=O)NC(=O)c2cc3n4c(-c5c(cc(OC)cc5)C=C(C4)C(=O)N(CCCCN1C)C)c(c3cc2)C1CCCCC1	42	4.35	590	100.955	9	1

[a] Molecular polar surface area; [b] Number of H-bond acceptor atoms; [c] Number of H-bond donor atoms.

1. RMSD Values for Each Conformer

The conformers were generated with molecular dynamics/large-scale low-mode sampling (MD/LLMod) method [1]. For each conformer, the calculated RMSD (with respect to the crystal structure) is given in the graphics that follow:

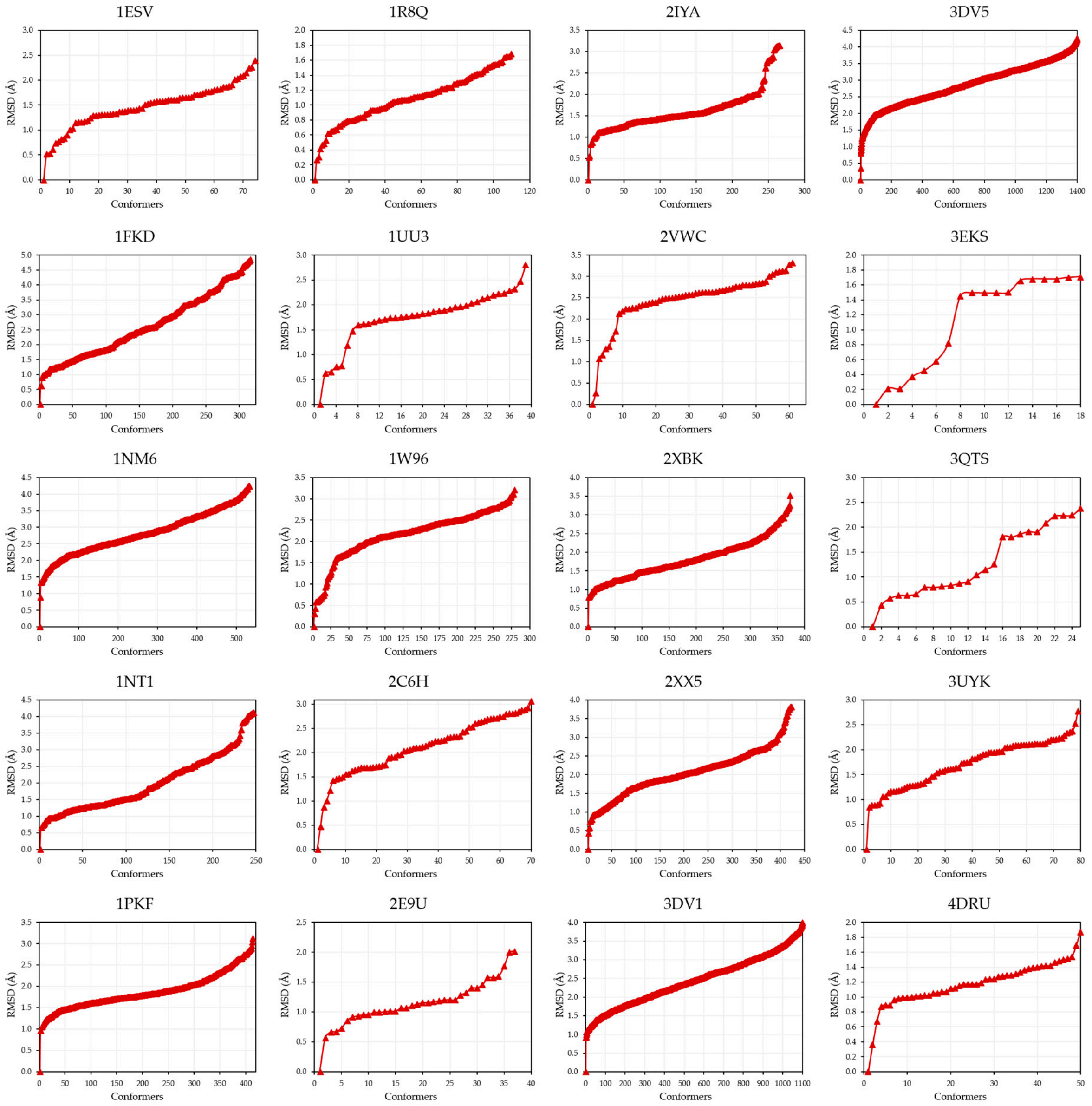

Table S3. Conformational statistics.

			RMSD	RMSD	95% CI (Å)[a]		RMSD	95% CI (Å)[b]	
		**No. conf.**	**mean**	**SD**	**(−)**	**(+)**	**median**	**(−)**	**(+)**
**1**	1ESV	73	1.47	0.43	1.37	1.57	1.54	1.44	1.64
**2**	1FKD	315	2.63	1.08	2.51	2.75	2.53	2.41	2.65
**3**	1NM6	531	2.82	0.66	2.76	2.88	2.78	2.72	2.84
**4**	1NT1	246	1.99	0.86	1.88	2.09	1.81	1.70	1.92
**5**	1PKF	412	1.87	0.41	1.83	1.91	1.80	1.76	1.84
**6**	1R8Q	109	1.08	0.33	1.02	1.15	1.10	1.04	1.16
**7**	1UU3	38	1.74	0.55	1.57	1.91	1.82	1.65	1.99
**8**	1W96	278	2.17	0.57	2.10	2.23	2.25	2.18	2.32
**9**	2C6H	69	2.09	0.59	1.95	2.23	2.13	1.99	2.27
**10**	2E9U	36	1.14	0.38	1.01	1.26	1.13	1.01	1.25
**11**	2IYA	265	1.62	0.49	1.56	1.68	1.51	1.45	1.57
**12**	2VWC	60	2.44	0.63	2.28	2.60	2.57	2.41	2.73
**13**	2XBK	373	1.81	0.55	1.76	1.87	1.75	1.69	1.81
**14**	2XX5	422	2.05	0.64	1.99	2.12	2.04	1.98	2.10
**15**	3DV1	1106	2.45	0.68	2.41	2.49	2.44	2.40	2.48
**16**	3DV5	1402	2.88	0.65	2.84	2.91	2.91	2.88	2.94
**17**	3EKS	17	1.12	0.62	0.83	1.41	1.49	1.20	1.78
**18**	3QTF	24	1.27	0.68	1.00	1.54	1.04	0.77	1.31
**19**	3UYK	78	1.71	0.48	1.60	1.81	1.82	1.71	1.93
**20**	4DRU	50	1.18	0.32	1.10	1.27	1.17	1.08	1.26

[a] 95% confidence interval of the mean; [b] 95% confidence interval of the median.

2. The C7 Stereocenter of 9 (YC-17, Complex 2C6H)

The absolute configuration of ligand **9** (compound YC-17), which was subjected to the molecular dockings explained in the main text, is that found in crystal structure PDB 2C6H, shown in Figure S1A (7*S*). However, the original report of this complex [2] and other papers concerning this molecule [3] indicate that in the natural product the configuration of C7 is *R*, as shown in Figure S1B.

Where is the mistake? Was epimer 7*S* (Figure S1A) subjected to co-crystallization by the authors? Or was the natural product (7*R*, Figure S1B) subjected to co-crystallization but the epimerization took place in the meantime (e.g., due to the presence of a tertiary amine in the structure)?

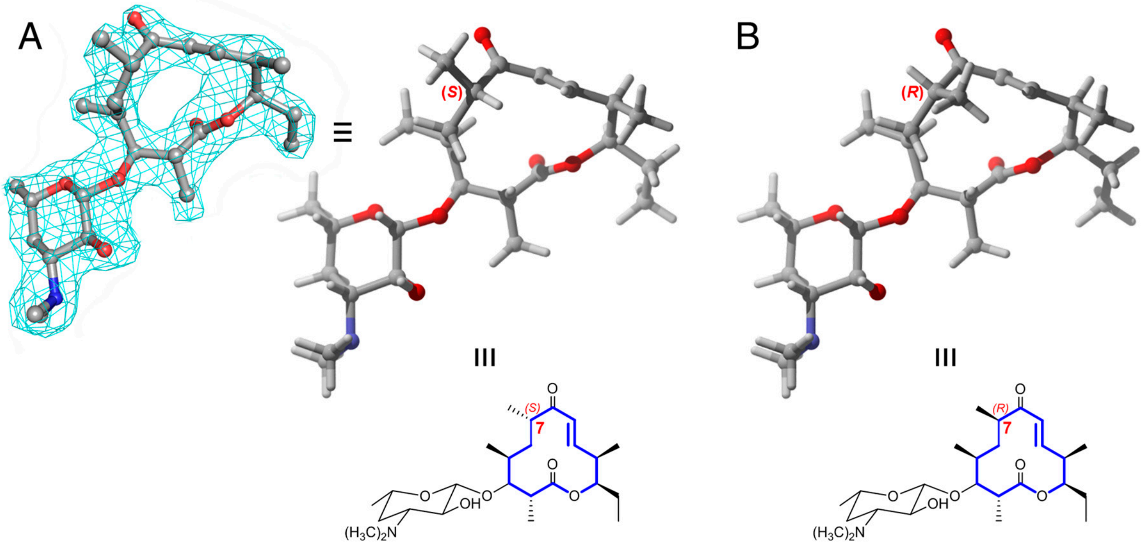

Figure S1. (**A**) Ligand **9** with the density map downloaded from the Electron Density Server web (http://eds.bmc.uu.se/); (**B**) compound **9** as it appears in the chemical literature [2,3].

To estimate the relevance of the configuration at C7, we carried out representative dockings with epimer 7*R* (**B**) by using AD Vina and Glide. The results (second line of the following summary) were compared with those in Table 4 of the manuscript (first line of the following summary):

		AD Vina			Glide		
		**RMSD**	**Score**	**Pose Order**	**RMSD**	**Score**	**Pose Order**
**9** (7*S*) in 2C6H	Table 4 & Figure S1A	0.56	−10.8	2	0.82	−6.85	15
epi-**9** (7*R*)	Figure S1B	0.79	−9.4	1	0.79	−7.12	2

The result of Glide suggests that the natural product (B) fits better in the binding pocket. A mistake in the PDB drawing is possible.

3. RMSD$_{overlap}$ (See RMSD Overlap, Page 8 of the Main Text)

For each ligand, the more reliable poses in Tables 5 and 6 of the main text were superimposed with the experimental pose (crystalline structure). The RMSD of the overlaps are given below.

Re-scoring with AD 4.2//Vina (Table 5, right). Comparison with the corresponding crystalline structures:

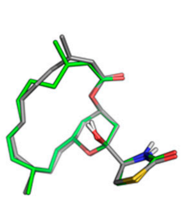

1
RMSD$_{overlap}$ 0.38

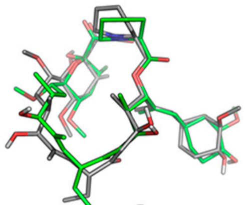

2
RMSD$_{overlap}$ 0.87

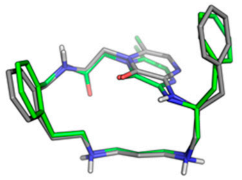

3
RMSD$_{overlap}$ 0.67

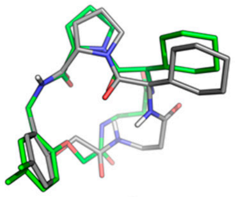

4
RMSD$_{overlap}$ 0.79

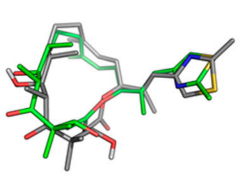

5
RMSD$_{overlap}$ 1.04

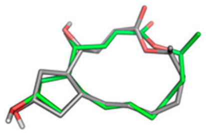

6
RMSD$_{overlap}$ 0.83

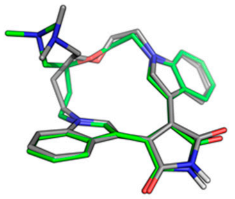

7
RMSD$_{overlap}$ 0.65

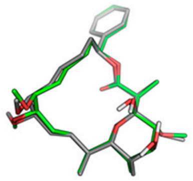

8
RMSD$_{overlap}$ 0.32

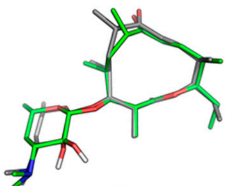

9
RMSD$_{overlap}$ 0.53

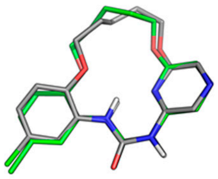

10
RMSD$_{overlap}$ 0.67

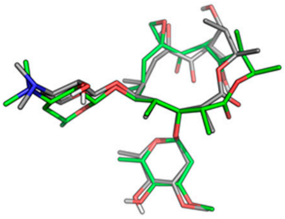

11
RMSD$_{overlap}$ 1.05

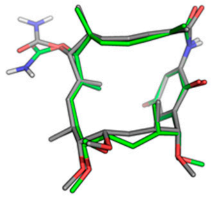

12
RMSD$_{overlap}$ 0.63

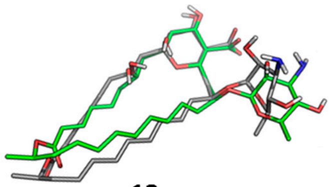

13
RMSD$_{overlap}$ 1.08

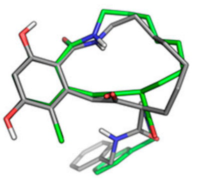

14
RMSD$_{overlap}$ 1.10

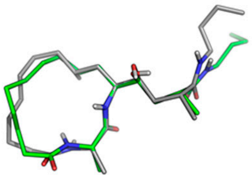

15
RMSD$_{overlap}$ 0.86

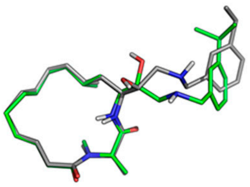

16
RMSD$_{overlap}$ 0.93

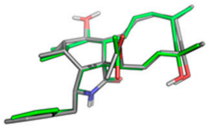

17
$RMSD_{overlap}$ 0.61

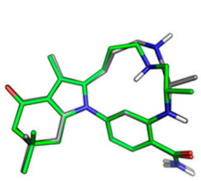

18
$RMSD_{overlap}$ 0.42

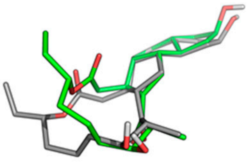

19
$RMSD_{overlap}$ 2.14

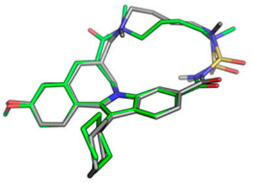

20
$RMSD_{overlap}$ 0.85

Figure S2. Structures of the docked ligands (AD 4.2//Vina) are depicted in grey and those of the ligands in the crystal in green.

Re-scoring of DOCK with Amber (Table 6, up). Comparison with the corresponding crystalline structures:

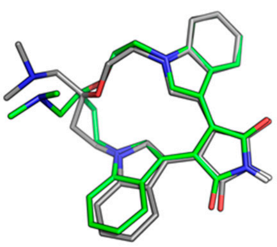

7
$RMSD_{overlap}$ 0.87

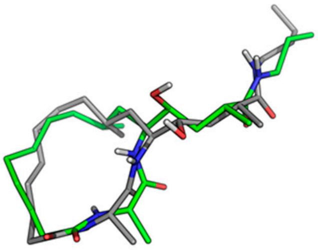

15
$RMSD_{overlap}$ 1.12

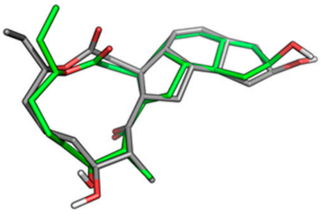

19
$RMSD_{overlap}$ 0.75

Figure S3. Docked ligands (Amber from DOCK) drawn in grey; experimental poses of the ligands (crystal structures) in green.

Re-scoring of Glide with MM-GBSA (Table 6). Comparison with the corresponding crystalline structures:

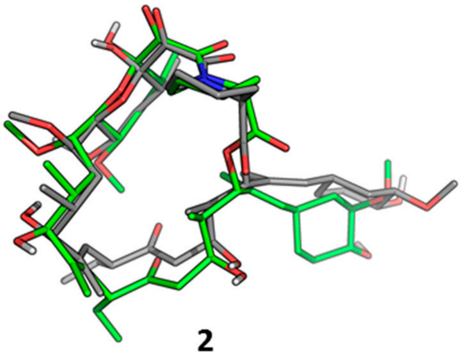

2
$RMSD_{overlap}$ 1.13

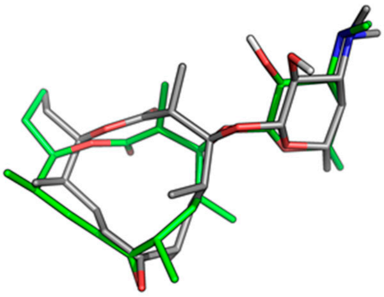

9
$RMSD_{overlap}$ 1.36

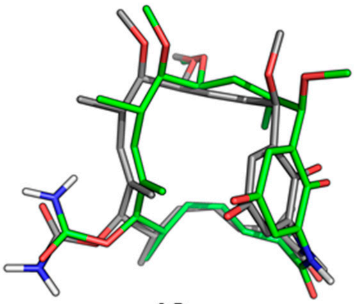

12
$RMSD_{overlap}$ 0.69

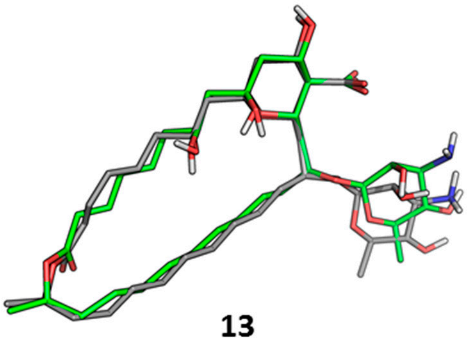

13
$RMSD_{overlap}$ 0.64

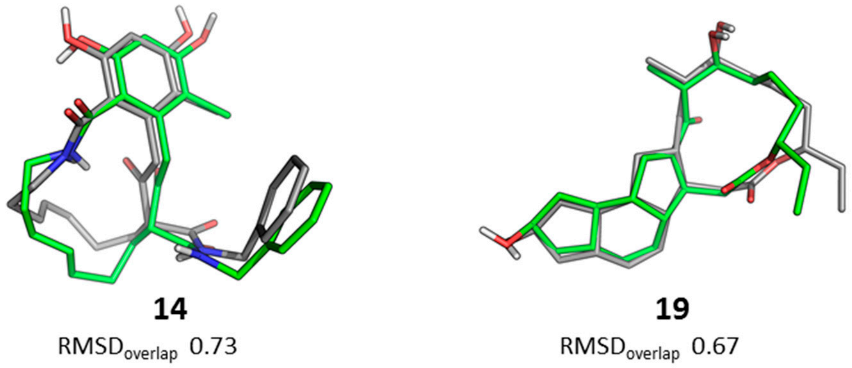

Figure S4. Docked ligands (MM-GBSA from Glide) depicted in grey; experimental poses in green.

4. AutoDock 4.2 vs. AutoDock 4.2 Flex (Flexible Rings Docking)

One of the advantages of AD 4.2 is that it permits to take into account the flexibility of the rings during the docking time [4]. However, the dockings carried out with the flexible rings subroutine (AD 4.2 Flex) did not improve the standard results (Table S2), as shown in Table S4, except for ligands **14** and **19**.

Table S4. Comparison of AD 4.2 with AD 4.2 Flex. [a]

NO.	PDB ID	AD 4.2 RMSD	AD 4.2 Flex RMSD
**1**	1ESV	0.72	**6.10**
**2**	1FKD	1.09	**4.63**
**3**	1NM6	0.89	**6.22**
**4**	1NT1	1.33	1.25
**5**	1PKF	1.59	1.86
**6**	1R8Q	1.23	1.12
**7**	1UU3	1.27	1.40
**8**	1W96	0.69	1.53
**9**	2C6H	**3.94**	**4.51**
**10**	2E9U	0.79	0.95
**11**	2IYA	0.98	1.24
**12**	2VWC	0.79	**5.98**
**13**	2XBK	1.72	2.01
**14**	2XX5	**6.41**	**1.50**
**15**	3DV1	1.14	**3.57**
**16**	3DV5	1.10	**6.39**
**17**	3EKS	0.71	1.24
**18**	3QTF	1.28	0.32
**19**	3UYK	**4.88**	**0.80**
**20**	4DRU	1.31	0.65
**Mean**		**1.69**	**2.66**

[a] The highest (poorest) RMSD values are indicated in bold red.

Three representative examples are depicted below. In case A the flexible rings docking produced a result similar to the standard AD 4.2. In B the RMSD values using AD 4.2 Flex increased too much, which was quite common. In C the RMSD values obtained with AD 4.2 Flex improved with regard to the AD 4.2 values (this occurred for cases **14** and **19**, as mentioned above).

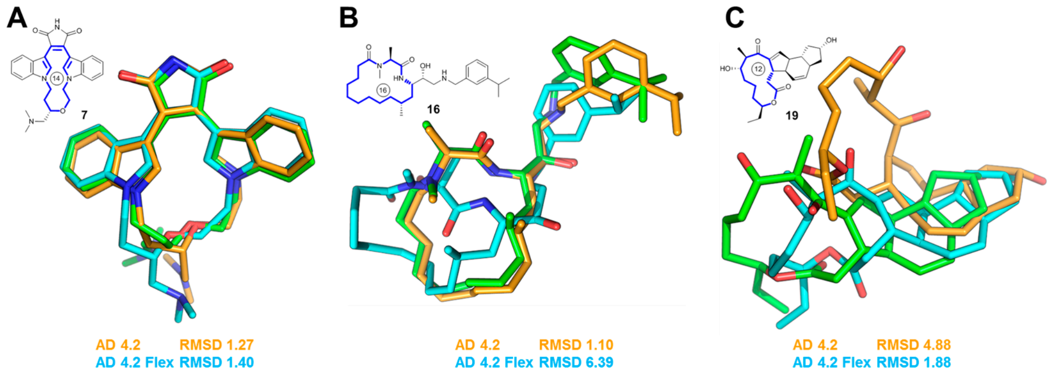

Figure S5. Structures of 7 (**A**); 16 (**B**); and 19 (**C**). The co-crystallized ligands are drawn in green. The ligands as docked with AD 4.2 Flex are in cyan. Those docked with AD 4.2 are depicted in orange.

If only the macrolide subset (**1**, **5**, **6**, **8**, **9**, **11**, **13**, and **19**) is considered, there is one case (**1**) in which the use of AD 4.2 Flex was very detrimental and another (**19**) in which it was clearly beneficial.

References

1. Watts, K.S.; Dalal, P.; Tebben, A.J.; Cheney, D.L.; Shelley, J.C. Macrocycle Conformational Sampling with MacroModel *J. Chem. Inf. Model.* **2014**, *54*, 2680–2696.
2. Sherman, D.H.; Li, S.; Yermalitskaya, L.V.; Kim, Y.; Smith, J.A.; Waterman, M.R.; Podust, L.M. The Structural Basis for Substrate Anchoring, Active Site Selectivity, and Product Formation by P450 PikC from Streptomyces venezuelae *J. Biol. Chem.* **2006**, *281*, 26289–26297.
3. Shinde, P.B.; Han, A.R.; Cho, J.; Lee, S.R.; Ban, Y.H.; Yoo, Y.J.; Kim, E.J.; Kim, E.; Song, M.-C.; Park, J.W.; Lee, D. G.; Yoon, Y. J. Combinatorial biosynthesis and antibacterial evaluation of glycosylated derivatives of 12-membered macrolide antibiotic YC-17 *J. Biotechnol.* **2013**, *168*, 142–148.
4. Forli, S.; Botta, M. Lennard-Jones Potential and Dummy Atom Settings to Overcome the AUTODOCK Limitation in Treating Flexible Ring Systems *J. Chem. Inf. Model.* **2007**, *47*, 1481.
